# Local Drug Delivery for the Treatment of Neurotology Disorders

**DOI:** 10.3389/fncel.2019.00238

**Published:** 2019-06-03

**Authors:** Fabrice Piu, Kathie M. Bishop

**Affiliations:** Otonomy Inc., San Diego, CA, United States

**Keywords:** drug delivery, local, inner ear, neurotology disorders, intratympanic administration

## Abstract

Neurotology disorders such as vertigo, tinnitus, and hearing loss affect a significant proportion of the population (estimated 39 million in the United States with moderate to severe symptoms). Yet no pharmacological treatments have been developed, in part due to limitations in effective drug delivery to the anatomically protected inner ear compartment. Intratympanic delivery, a minimally invasive injection performed in the office setting, offers a potential direct route of administration. Currently, off-label use of therapeutics approved to treat disorders via systemic administration are being injected intratympanically, mostly in the form of aqueous solutions, but provide variable levels of drug exposure for a limited time requiring repeated injections. Hence, current drug delivery approaches for neurotology disorders are sub-optimal. This review, following a description of pharmacokinetic considerations of the inner ear, explores the merits of novel delivery approaches toward the treatment of neurotology disorders. Methodologies employing local delivery to the inner ear are described, including direct intracochlear delivery as well as intratympanic methods of infusion and injection. Intratympanic injection delivery formulation strategies including hydrogels, polymers and nanoparticulate systems are explored. These approaches represent progress toward more effective delivery options for the clinical treatment of a variety of neurotology disorders.

## Introduction

Neurotology disorders of the inner ear such as hearing loss, vertigo, and tinnitus affect a large proportion of the population with significant impact on patient’s quality of life (National Institute of Health, 2000). It is estimated that, in the United States alone, 1 in 8 individual (or about 39 million) suffers from moderate to severe hearing loss, tinnitus or vertigo (Neitzel et al., 2017). Inner ear disorders of cochlear origin most commonly manifest themselves clinically as hearing loss due to a number of factors such as age-related hearing loss (presbycusis), ototoxicity-related hearing loss (i.e., due to certain classes of chemotherapeutics or antibiotics), noise induced hearing loss (NIHL), sudden sensorineural hearing loss (SSNHL), and genetic forms of hearing loss. The most commonly diagnosed vestibular disorders include benign paroxysmal positional vertigo (BPPV), labyrinthitis or vestibular neuritis. In addition, a confluence of symptoms is evident in disorders such as Meniere’s disease where patients experience vertigo, hearing loss, tinnitus, and aural fullness, and tinnitus itself is a debilitating symptom accompanying many forms of hearing loss (D’aldin et al., 1999; Gupta and Sataloff, 2003; Swartz and Longwell, 2005; Harris and Salt, 2007; Liu and Yan, 2007; Panda et al., 2008; Chan, 2009). The medical management of these disorders has largely focused on systemic delivery of drugs, surgical intervention, device use (e.g., hearing aids and cochlear implants) and behavioral therapy (Barritt, 2008). However, these approaches vary in their effectiveness and thus significant unmet need for the treatment of inner ear disorders exists.

Pharmacologic treatment of inner ear disorders such as hearing loss, tinnitus, and vertigo disorders has been challenging due to poor drug availability to this protected compartment with systemic administration. A shift is occurring toward the implementation of novel technologies and local routes of administration (Barritt, 2008; Swan et al., 2008; Hu and Parnes, 2009; McCall et al., 2009). Primarily, it is the result of the recognition that systemic routes of administration for inner ear therapy are severely limited due to poor drug exposure to the otic compartment due to the blood-labyrinth barrier, and significant risks of undesirable side effects with systemic delivery. This review explores and discusses novel approaches being developed for the therapeutic management of otic disorders. These technologies focus on local delivery directly to the middle ear, via passive absorbption through the round window membrane (intratympanic) or through other minor routes (diffusion through the oval window, bony channels, fissula and fenestrum) to the inner ear, or directly to the inner ear (intracochlear). A special emphasis is given to intratympanic injection, a minimally invasive drug delivery approach, and formulation strategies for intratympanic injection including hydrogels, polymers and nanoparticulate systems because of their convenience of use and potential to deliver therapeutic levels of drug over an extended period of time.

## Pharmacokinetic Considerations of the Inner Ear

**FIGURE 1:**
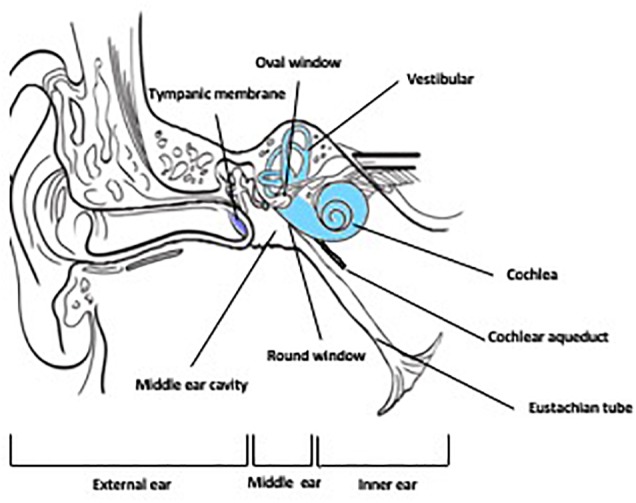
Anatomy of the ear.

The ear, the sensory organ comprising the auditory system (sound processing) and the vestibular system (balance and equilibrium) is anatomically and functionally divided into three regions: the outer ear, middle ear and the inner ear (Figure 1). The external ear is the external portion of the organ whose function is to collect and direct sound waves toward the tympanic membrane and the middle ear. The middle ear, an air-filled hollow called the tympanic cavity, is located behind the tympanic membrane and comprised of bony and ligament structures (auditory ossicles and stapes) providing a mechanical linkage between the tympanum and the inner ear for the transmission of sound waves. The inner ear is a fluid-filled compartment and the core organ for auditory signal transduction. It consists of two major compartments: the cochlea where auditory signal processing takes place and the vestibular where balance is modulated. The inner ear is a complex network of fluid-filled tubes known as the bony otic capsule and composed of two compartments with membranous barriers, one filled with perilymph, the other with endolymph. The bony otic capsule is the major anatomic and physiological barrier to inner ear drug delivery. The cochlea comprises a highly specialized structure, the organ of Corti, that contains the mechano-sensory cells of the inner ear (hair cells). Two structures separate the middle ear and the inner ear. The round window membrane (RWM) is a semi permeable membrane composed of three layers: outer epithelial layer facing the middle ear, a middle connective layer, and an inner cellular layer. Its permeability is known to be affected by many factors under normal and pathological conditions (Goycoolea, 1992). The other structure is the oval window, which is covered by the footplate of the stapes in the middle ear.

Because of its anatomical complexity, the pharmacokinetics of the inner ear are multifaceted. The large fluid-filled extracellular spaces that comprise it have multiple interconnections, in addition to interfacing with outside compartments such as the systemic blood circulation, the cerebrospinal fluid, and the middle ear cavity (via the oval and round window membranes). In general, the pharmacokinetic processes in the inner ear follow the established ADME principles but are centered on inner ear fluids rather than blood circulation (Table 1; Plontke and Zenner, 2002; Salt and Plontke, 2005, 2009; Salt, 2006).

**Table 1:** Pharmacokinetics of the inner ear.

	Systemic delivery	Local delivery
Absorption	Via stria vascularis and blood labyrinth barrier	Via round window, oval window and bony otic capsule
Distribution	Into inner ear fluid spaces, inner ear tissues; driven by passive diffusion	
Metabolism	Via protein binding, enzymatic processes	
Excretion	Via loss to fluid spaces in the cochlea, uptake into intercellular spaces, loss to cochlear bloodstream, loss to the cerebrospinal fluid via the cochlear aqueduct, and to the middle ear via the round window and oval window membranes	

**FIGURE 2:**
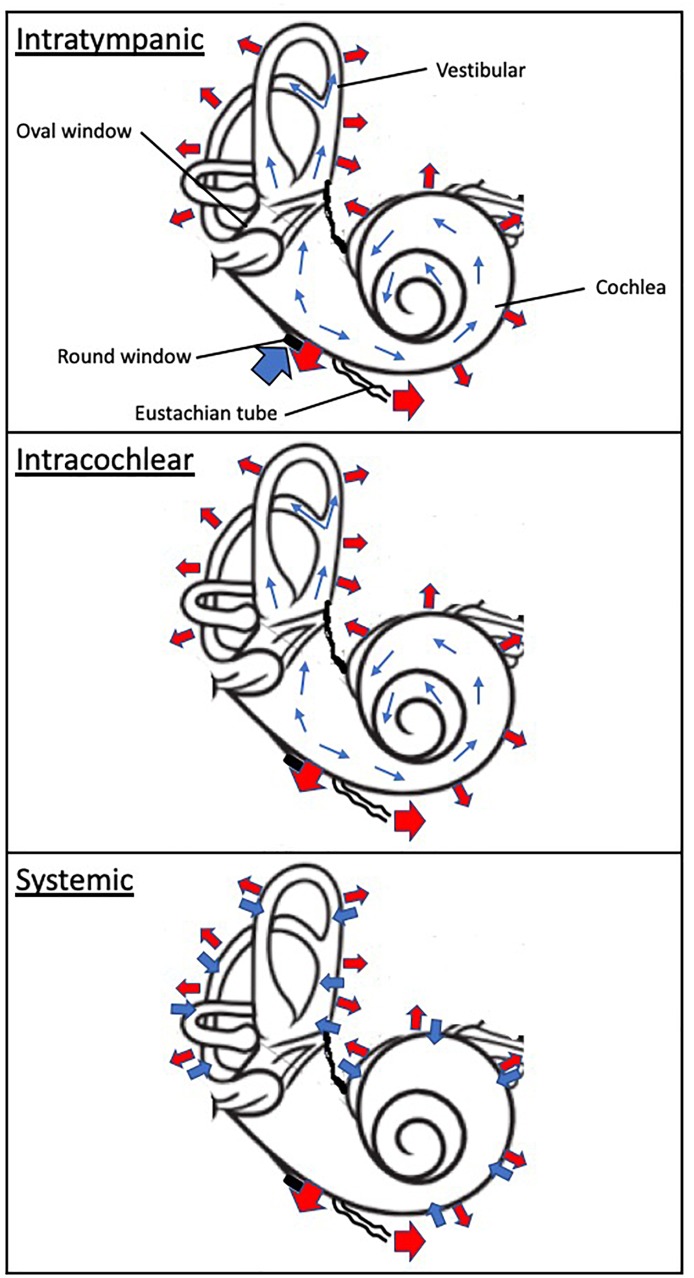
Schematic of drug absorption and elimination following intratympanic, intracochlear, and systemic administration. The wide blue arrows indicate drug absorption to the inner ear compartment, including the cochlea and vestibular. The thin blue arrows denote distribution within the inner drug compartment. The wide red arrows depicts elimination from the otic compartment through (1) the inner ear blood circulatory system which interfaces throughout the cochlea and vestibular apparatus, (2) loss to the cochlear aqueduct and (3) loss to the middle ear. The black disk symbolizes the round window.

Absorption of drug substances in the inner ear is largely dependent of the route of administration (Figure 2). Following systemic administration, pharmacological agents enter the inner ear via the stria vascularis, an area of capillary loops and small blood vessels located at the upper portion of the cochlear spiral ligament. This structure is surrounded by a network of endothelial cells connected via tight junctions, protecting the cochlea from the systemic circulation, and known as the blood-labyrinth barrier (BLB) (Juhn et al., 2001). Experimental studies have demonstrated that the BLB is an effective structure in limiting diffusion from the bloodstream into the cochlea, with rates of entry on average 4–6% of the total plasma concentration (Inamura and Salt, 1992). The BLB exhibits some functional attributes comparable to the blood-brain barrier (BBB), but also some differences. The BLB largely prevents the passage of substances from the blood into the inner ear, is seemingly less permeable to several ions (sodium, calcium, and calcium) than the BBB and differentially modulates the passage of larger substances as a function of the molecular weight compared to the BBB (Juhn et al., 2001). These differences in functionality are ascribed in part to the tight junction composition, permeability enhancers and receptors mediating endocytosis or transcytosis making up the BLB (Nyberg et al., 2019). Hence, the physicochemical properties of systemically administered drug will drive their diffusion across the BLB. As a result, drug exposure to the inner ear following systemic administration is limited and highly variable. For instance, Bird and colleagues noted a large inter-variability spanning three orders of magnitude in perilymph concentrations between human patients after systemic administration of a methylprednisolone aqueous solution (Bird et al., 2007) or a dexamethasone solution (Bird et al., 2011).

In contrast, following intratympanic administration, local absorption from the middle ear takes place via the round window, the oval window and the bony otic capsule. The RWM is considered a primary passage of intratympanic drugs but also a physical barrier to inner ear delivery. The oval window may be a promising delivery route to target the vestibular system and associated disorders. These structures provide access primarily to the cochlea, and through a diffusion process to the vestibular apparatus (Goycoolea, 2001; Imamura and Adams, 2003; Banerjee and Parnes, 2004). The round window membrane (RWM) is the most accessible of these structures; absorption of drugs through the RWM is dependent upon permeability properties and the contact duration of the applied drug substances. For instance, RWM permeability is markedly affected by the presence of additives such as membrane permeation enhancers (Li et al., 2018) and preservatives (Mikulec et al., 2008), certain attributes of the pharmacological agent such as molecular weight (Chelikh et al., 2003) and chemical properties (Salt et al., 2018; Salt and Plontke, 2018). Animal studies as well as computer simulations have demonstrated that prolonging the drug contact duration of aqueous solutions with the round window membrane results in increased drug levels in the inner ear (Plontke and Salt, 2006; Plontke et al., 2007a). Transport across the RWM relies on additional processes such as passive diffusion and active transport. While it has been suggested that the oval window offers an alternate route of absorption to the inner ear, its contribution and impact remain to be ascertained (Tanaka and Motomura, 1981; Takumida and Anniko, 2004). It has been proposed that drug substances could passively diffuse through the thin bone structure of the stapes and the walls of the oval window niche. Drug diffusion to the apex of the cochlea has been demonstrated to be possible in rodents through the bony otic capsule (Mikulec et al., 2008). However, the anatomical differences between rodents and humans, in particular the fact that the human cochlea is largely embedded in the temporal bone, suggest that this route of entry may be is significantly more challenging in humans.

Distribution of drugs in the inner ear is mostly governed by passive diffusion (Figure 2). Unlike the circulatory system, the perilymphatic and endolymphatic fluids of the inner ear display extremely slow rates of volume flow (Salt et al., 1986; Ohyama et al., 1988; Salt and Ma, 2001). Because passive diffusion is a non-linear process over distance, gradients along the length of the cochlea are observed when drugs are deposited at the base of the cochlea (round window niche) (Salt et al., 1986; Plontke et al., 2007a). Additionally, drugs passively diffuse into tissues and fluid spaces (Salt et al., 1991a,b). This interscalar distribution contributes to a net loss of drug to the cochlear blood flow the magnitude of which is believed to be significant (Salt and Plontke, 2005). Since the inner ear distribution of substances is primarily driven by concentration gradients, any changes in these gradients will affect drug availability to the local compartment in a process coined “redistribution.” Finally, studies of the perilymph suggest that protein binding of acidic and lipophilic drugs could alter drug distribution in the perilymph by trapping pharmacological agents (Swan et al., 2009).

The elimination or clearance of drugs in the inner ear fluids is primarily driven by metabolism, which is dependent upon the nature of the drugs themselves. Building on the initial study by Thalmann et al. (1994), it was recently demonstrated that the perilymphatic compartment contains proteins such as albumin, alpha-1 acid protein, lipoproteins as well as enzymes (Swan et al., 2009). Protein binding to drug substances would protect them from being metabolized rapidly, while serving as drug reservoirs and carriers. To date, only a few studies have investigated the enzymatic processes taking place in inner fluids. Examples illustrate that the prodrug dexamethasone-phosphate is converted into its active moiety dexamethasone (Mikulec et al., 2008), and the activity of purines is modulated by ectonucleosidases (Chelikh et al., 2003). Other clearance mechanisms have been described, including loss to the fluid spaces in the cochlea, uptake into intercellular spaces, loss to the cerebrospinal fluid via the cochlear aqueduct, and to the middle ear via the round window and oval window membranes (Salt and Plontke, 2009).

## Inner Ear Drug Delivery

Systemic delivery may seem to be the preferred approach for therapeutic treatment of inner ear disorders because of its convenience. However, systemic routes of administration (such as oral and intravenous) may result in limited therapeutic access to inner ear target structures, with drug levels being several orders of magnitude lower than following local intratympanic administration (Bird et al., 2007, 2011). The protection imparted by blood-labyrinth barrier against the diffusion of substances from the circulatory system into the inner ear, the different metabolic pathways and various routes of excretion, all contribute to a variable fate of the drug in the inner ear, hence possibly resulting in variable clinical outcome (Paulson et al., 2008). Consequently, dosing regimens have aimed at delivering high drug levels systemically in the hope of achieving adequate inner ear therapeutic efficacy, creating potential systemic side effect liabilities.

Because of the limitations of systemic delivery, alternative means to efficiently deliver medications to the inner ear have been explored. Since the initial work by Ersner et al. (1951) and decades later by others (Sakata et al., 1986; Shea and Ge, 1996) demonstrating the clinical potential of locally administered drugs in the ear, there has been a trend in clinical practice to recognize and implement local delivery procedures as a more effective approach (Hu and Parnes, 2009; MCcall et al., 2010).

Comparison of different approaches for drug delivery to the inner ear compartment is summarized in Table 2.

**Table 2:** Comparison of different routes of administration to the inner ear.

	Benefits	Limitations
Systemic	- Non-invasive - Ease of administration	- Limited inner ear exposure due to poor drug absorption across BLB - Large systemic exposure with potential for dose limitation, and associated risks of adverse effects
Intracochlear	- Direct exposure to the inner ear - Prolonged and controlled cochlear drug delivery via perfusion/devices - Limited systemic exposure	- Invasive (surgical procedure required) - Risks of infection of the inner ear - Device performance restrictions
Intratympanic Extracochlear implants	- Significant exposure to the inner ear - Prolonged and controlled drug delivery via perfusion/devices - Limited systemic exposure	- Required drug diffusion across the RWM into inner ear - Moderately invasive (incision of the ear drum) - Risks of persistent ear drum perforation, ear infection - Risks of hearing degradation
Intratympanic Natural polymers Synthetic polymers Nanoparticulates	- Minimally invasive - Significant exposure to the inner ear - Limited systemic exposure	- Required drug diffusion across the RWM into inner ear - Depending on polymer, may require several injections due to limited duration of exposure - Depending on polymer, risks of middle ear inflammation/fibrosis
Intratympanic Poloxamer	- Minimally invasive - Significant and prolonged exposure to the inner ear (weeks to months) following a single administration - Limited systemic exposure	- Required drug diffusion across the RWM into inner ear

### Intracochlear Delivery

Intracochlear delivery devices are currently being developed to deliver drugs directly to the inner ear. These are largely modifications of existing technologies such as cochlear implants, osmotic pumps and microperfusion systems surgically implanted into the inner ear. By modifying cochlear implant electrodes and coupling them to an external pump system, Paasche et al. (2006) demonstrated the possibility of delivering a bioactive molecule directly to the inner ear. The coating of cochlear electrodes with a biopolymer loaded with a bioactive agent has also been investigated and demonstrates encouraging preliminary findings (Hendricks et al., 2008; Richardson et al., 2009).

Controlled delivery devices can offer the benefit of managing the rate and duration of drug release. Advance of these technologies is dependent upon the miniaturization and integration of multiple functions in a single implant. To date, some osmotic pumps have been approved for non-otic medical use, but are of limited benefit because of non-adjustable dosing regimen, limited duration of drug delivery, and no on/off control of the pump’s actions (MCcall et al., 2010). Microfluidic systems, on the other hand, incorporate flexibility of programming drug infusion rates. Advances have been made recently in this area leading to the development of a self-contained, programmable device (Chen et al., 2005; Fiering et al., 2009; Sewell et al., 2009; Peppi et al., 2018). Cochlear infusion of a glutamate receptor antagonist in guinea pigs was achieved for 30 days using a microfluidic system (Fiering et al., 2009). More recently, an open label study was conducted in tinnitus patients under a compassionate treatment protocol. The NMDA receptor antagonist gacyclidine was administered intracochlearly via a DurectRWmuCath^TM^ into the round window niche for a period of 3–4 days (Wenzel et al., 2010). Four of the six treated patients demonstrated a transient improvement in their tinnitus perception. Another human study explored the safety of dexamethasone-eluting cochlear implant electrodes, and demonstrated lower impedances in these subjects (Plontke et al., 2016). In general, the most obvious drawback to these technologies is the requirement for surgical implantation and potential for device performance issues once implanted.

### Intratympanic Delivery

The interconnections between the cochlea and the vestibular structures enable any pharmacological agent administered intratympanically to potentially reach, via the round window, all of the inner ear substructures. Thus, tractable delivery is possible via intratympanic administration, especially in humans where the inner ear is embedded into the skull near the brainstem, resulting in extreme inaccessibility except via an intratympanic approach.

#### Implantable Extracochlear Catheters

One of the first attempts at developing a sustained release system for delivery onto the RWM was the MicroWick (Silverstein, 1999). This device consists of a polyvinyl acetate wick (1 mm in diameter by 9 mm in length) applied to the round window niche through a ventilation tube placed in the tympanic membrane (myringotomy). Patients would then self-administer the medication into the external canal. A typical dosing regimen requires multiple daily applications for several weeks. Since its inception, a few studies have been published demonstrating the effectiveness of the Microwick in the treatment of Meniere’s disease using gentamicin (Hill et al., 2006; Suryanarayanan et al., 2009), SSNHL using steroids (Herr and Marzo, 2005; Van Wijck et al., 2007) and AIED using a TNFα blocker (Van Wijk et al., 2006). In the Meniere’s disease studies, long term vertigo control with gentamicin was observed in the majority of patients at the 24-month follow-up. In the SSNHL studies, significant improvement in hearing (∼25 dB) as measured by pure tone average was observed in 8 out of 12 patients. The AIED study reported that the delivery of the TNFα blocker once weekly for 4 weeks resulted in hearing improvement and reduction in disease relapse. However, complications from the Microwick were observed in small fraction of patients (∼25%) and consisted of a persistent perforation of the tympanum, ear infection, the development of fibrosis in the middle ear, and hearing degradation (Herr and Marzo, 2005; Suryanarayanan et al., 2009).

Round window microcatheters, such as μ-Cath and e-Cath, consist of several lumens: a first one for drug administration, a second one for middle ear fluid drainage, and sometimes a third one incorporating an electrode to monitor ear signals (MCcall et al., 2010). Implantable catheters are anchored in the round window bony niche and protrude through the tympanic membrane into the external ear canal. Drug is usually administered using a microperfusion pump device, which ensures continuous drug delivery at a constant flow rate over a period of several weeks. Several clinical studies using microcatheters in the treatment of Meniere’s disease (Schoendorf et al., 2001; Plontke et al., 2009) and SSNHL (Kopke et al., 2001; Lefebvre and Staecker, 2002; Herr and Marzo, 2005; Plontke et al., 2005, 2006) have been published, almost exclusively with steroids. Two non-randomized pilot studies delivered steroids to SSNHL patients who failed oral steroids; improvements in hearing and speech discrimination were observed in a majority of patients. A randomized placebo control study of similar design demonstrated a tendency toward better hearing in the treatment group, but did not reach statistical significance. Overall, success in clinical outcome has been variable but noted improvements in clinical endpoints were seen for the majority of the patients. Similarly to the Microwick, implantable catheters can be associated with catheter dislocation and/or obstruction, persistent tympanic perforation and ear infection (Plontke et al., 2005).

#### Intratympanic Injection

Current intratympanic injection approaches focus on the off-label use of approved drug formulations that were initially formulated and developed for intravenous administration. An empirical approach has been employed to define the dose selection and dosing schedule in the off-label clinical use of these drugs for inner ear therapy. For instance, corticosteroids have been widely used in the treatment of Meniere’s disease (Silverstein et al., 1998, 2009; Araujo et al., 2005; Garduno-Anaya et al., 2005; Kitahara et al., 2008a) and SSNHL (Chandrasekhar, 2001; Kopke et al., 2001; Xenellis et al., 2006; Battaglia et al., 2008; Plontke et al., 2009) Dosing schedules and regimen vary considerably between clinicians, with the total dose received differing by more than two orders of magnitude and with intratympanic injections given as often as once daily for 10 consecutive days (Peng et al., 2008) or as rarely as a single injection (Haynes et al., 2007). Furthermore, patients are asked to remain in a supine position for up to an hour and counseled not to swallow, as these behaviors can lead to rapid elimination of solution out of the middle ear via the Eustachian tube. The confluence of these variables is an important factor in the reported variability in clinical outcomes observed among patients in these studies. Bird and colleagues noted a large inter-variability in perilymph concentrations between patients after intratympanic administration of a methylprednisolone (Bird et al., 2007) or a dexamethasone (Bird et al., 2011) solution, providing a rationale for the reported variable clinical successes. Several factors can positively influence the clinical outcome. More than the absolute dose given at each administration or the total cumulative dose, the number of injections and the interval between them emerge as better predictors of a positive clinical outcome. These findings appear to be valid across drug classes (dexamethasone, methylprednisolone) and disease modalities (MD, SSNHL). Therefore, a more effective approach resides in developing delivery systems that prolong the drug exposure in the inner ear compartment. In addition, such systems might minimize the risks associated with multiple intratympanic injections over a short period of time, including perforation of the tympanic membrane, ear infection and otorrhea.

## Novel Formulations for Inner Ear Delivery

The intratympanic delivery strategies discussed above underscores the need for developing more effective delivery systems that prolong the drug exposure in the inner ear compartment following a single administration, primarily by overcoming delivery barriers and improving drug retention. Such systems typically rely on the use of various classes of polymers to temporally modulate drug release. In addition, these approaches might minimize the risks associated with multiple intratympanic injections over a short period of time, including perforation of the tympanic membrane, ear infection and otorrhea. A summary of novel formulations for inner ear delivery that have entered clinical development is presented in Table 3.

**Table 3:** Novel formulations for intratympanic delivery that have entered clinical development.

Drug	Active pharmaceutical ingredient	Therapeutic class	Indication	Drug delivery system	Clinical status
OTIPRIO	Ciprofloxacin	Antibacterial	Otitis media with effusion undergoing tympanostomy tube placement Acute otitis externa	Poloxamer	FDA approved
OTIVIDEX	Dexamethasone	Steroid	Vertigo associated with Meniere’s disease	Poloxamer	Phase III
AM-101	S-Ketamine	NMDA antagonist	Tinnitus	Hyaluronic acid	Phase III
AM-111	D-JNKI-1	JNK inhibitor	Hearing loss	Hyaluronic acid	Phase III
FX-322	Progenitor Cell Activation	Several	Hearing loss	Poloxamer	Phase I/II
OTO-311	Gacyclidine	NMDA antagonist	Tinnitus	Poloxamer	Phase I

### Natural and Synthetic Polymers

Polymers are macromolecular networks that can serve as controlled release drug delivery vehicles. These systems have been developed for a number of therapeutic applications, and recently their potential for local delivery to the inner ear has been investigated [reviewed in Wise and Gillespie (2012), Liu et al. (2013), El Kechai et al. (2015)].

The mechanism of drug absorption and release can vary substantially between the different classes of polymers (Van Tomme et al., 2008). Gelatin, a natural polymer, can be modified to present a negatively or positively charged profile, therefore allowing the binding of drugs through polyion complexation. Hyaluronic acid, another natural polymer of anionic charge, can also serve as a drug carrier. Drug release from these classes of polymers occurs via enzymatic hydrolysis of the polymer (Nakagawa and Ito, 2007). In contrast, with other biopolymers such as alginate and chitosan, drugs are released through diffusion out of the matrix. The use of synthetic polymers can confer additional benefits, especially in-situ gelling properties. A biological trigger, such as temperature or pH, would ensure the self-assembly of the matrix, that is the transition from a solution to a gel form. In some instances, this process can be reversible such as with triblock copolymers including poloxamer 407 (Dumortier et al., 2006). Other polymers such as poly(lactic-co-glycolic acid) or PLGA are organized as nanoparticles, that release drug into intracellular compartments following an endocytic absorption process (Xu et al., 2009).

Gelatin, in the form of Gelfoam, has been administered to the middle ear of both laboratory animals (Endo et al., 2005; Iwai et al., 2006; Lee et al., 2007; Inaoka et al., 2009) and humans (Arriaga and Goldman, 1998; Kitahara et al., 2008b; Nakagawa et al., 2014). Because the isoelectric point of gelatin can be engineered to create a positively or negatively charged polymer, it is a potentially useful carrier for proteins and nucleic acids. Growth factors such as BDNF, IGF-1 and HGF delivered to the inner ear in gelatin hydrogels showed detectable levels in the perilymph for up to 1 week and were effective in improving hearing in an animal model of NIHL (Iwai et al., 2006; Inaoka et al., 2009). Open-label clinical studies of Gelfoam dexamethasone in patients with Meniere’s disease showed inconsistent results with one study finding limited benefits (Arriaga and Goldman, 1998) and a second reporting long-term hearing improvement as measured by pure tone average and speech discrimination (Kitahara et al., 2008b). Histological assessments in animals have noted the significant ototoxic liability of gelatin hydrogels, especially the presence of a severe acute inflammatory response and the development of fibrosis in the middle ear, but the toxic findings did not extend to the cochlear and vestibular structures (Sheppard et al., 2004; Kitahara et al., 2008b).

Several classes of drugs have been loaded into hyaluronate polymers, including dexamethasone, S-ketamine, gentamicin, and kinase inhibitors which have been investigated in animal models of hearing loss and tinnitus (Yildirim et al., 2005; Barkdull et al., 2007; Coleman et al., 2007; James et al., 2008). Unlike Gelfoam, hyaluronate polymers appear safe to use and are not associated with persistent middle ear inflammation nor fibrosis but only provide a few days of exposure (Kelly et al., 1999).

Hyaluronic acid (Healon) delivery of dexamethasone has been evaluated in several small, open-label clinical studies of Meniere’s disease (Shea and Ge, 1996; Shea, 1997; Silverstein et al., 1998; Selivanova et al., 2005) and SSNHL (Gouveris et al., 2005; Stenner et al., 2006) patients with positive benefits reported. A randomized placebo-controlled Phase II clinical study reported on the safety and efficacy of AM-101 (S-Ketamine in hyaluronic acid) in acute tinnitus patients (van de Heyning et al., 2014). AM-101 was administered as three intratympanic injections over the course of 3 days and resulted in improvement in some tinnitus measures (loudness, annoyance) in a subset of tinnitus patients; however, these results were not replicated in Phase III studies^1^. The dosing regimen used in these clinical study highlights the limited prolonged exposure benefit of such a delivery approach. AM-111 (D-JNK inhibitor peptide in hyaluronic acid) is being developed for the treatment of SSNHL. Preclinical studies have demonstrated the activity of AM-111 in various models of otoprotection (Eshraghi et al., 2018). The current clinical experience demonstrated in a randomized placebo-controlled Phase II study improvements in hearing and speech in a subpopulation of patients with severe to profound acute SSNHL, but not in the overall study population (mild to profound acute SNHL) (Suckfuell et al., 2014).

There are only a few published pre-clinical studies and no clinical studies evaluating alginate and chitosan matrices in otologic conditions. Both polymers appear to only yield a few days of drug exposure in the inner ear, typically less than a week. Alginate can be tailored to deliver a variety of substances from small molecules to proteins to cells (Tonnesen and Karlsen, 2002). Noushi et al. (2005) reported administration of alginate beads to the middle ear of guinea pigs does not produce signification tissue inflammation or fibrosis. Chitosan polymers can also accommodate various biological materials, because of the positively charged nature of the matrix. Its drug sustained release properties can be tailored primarily by altering the sensitivity to lysozyme degradation. The only animal study describing the drug release profile of chitosan polymers indicated a short period of drug exposure in the inner ear of mice, of 3 to 5 days (Paulson et al., 2008). Chitosan polymers were not found to be associated with toxicity in the inner ear of guinea pigs (Saber et al., 2009).

### Nanoparticulate Systems

Nanoparticulate systems ranging from silica-based materials to liposomes and nanogels (Pyykko et al., 2016) represent an additional approach to otic formulation of drugs. PLGA nanoparticles can encapsulate bioactive molecules of various physicochemical properties (Bala et al., 2004; Nakagawa and Ito, 2007). Drug delivery to the cochlea using PLGA has been investigated in guinea pigs (Tamura et al., 2005), where deposition in the middle ear allowed delivery of rhodamine to the cochlea within 10 min of administration and present for the duration of the study (2 h). Similar findings were made in chinchillas following application of PLGA encapsulated iron oxide nanoparticles (40 min post-application) (Ge et al., 2007). Lidocaine loaded PLGA particles provided sustained release into the inner ear of guinea pigs for a short period of approximately 3 days (Horie et al., 2010) with no significant inflammation of the middle ear mucosa. The use of nanoparticle systems for delivery of growth factors has recently been explored – Nerve Growth Factor conjugated to lipid-based crystalline nanoparticles (Bu et al., 2015) applied to the RWM of guinea pigs resulted in exposure to the inner ear, but lasting only several hours. To date, no nanoparticulate systems have been evaluated clinically for otic delivery.

### Poloxamer-Based Polymers

Poloxamers, a group of triblock copolymers, are a particularly versatile class of polymer for inner ear delivery. They differ largely from other classes of polymers due to their amphiphilic nature, unique self-assembly properties, thermoreversible attributes and versatility of composition which make poloxamers amenable to broad applications (Bodratti and Alexandridis, 2018). Poloxamers consist of ethylene oxide and propylene oxide blocks arranged in a tripartite PEO-PPO-PEO configuration (Van Tomme et al., 2008). These copolymers display amphiphilic properties which are highly dependent upon the number of PEO and PPO units. The size, lipophilicity and hydrophilicity properties of poloxamers can be widely tailored.

Poloxamers exhibit thermoreversible and mucoadhesive properties, which make them particularly suitable for intratympanic inner ear drug delivery. Above the critical gelation concentration, PEO-PPO-PEO polymers exhibit a temperature dependent transition from a solution to a gel state in water. The transition temperature is determined by the copolymer composition, especially the ratio of PPO to PEO and the molecular weight of the polymer and thus can be set at body temperature. Further, poloxamers share bioadhesive characteristics that can be enhanced by the addition of various solvents and ionic agents. The bioadhesiveness typically increases as a function of gel strength and is associated with increased residence time.

Poloxamers are effective drug delivery vehicles for bioactive substances in the inner ear, ranging from small molecules to large proteins. In preclinical studies, dexamethasone levels in the inner ear are sustained for several weeks following a single intratympanic administration of OTIVIDEX (OTO-104, a micronized dexamethasone loaded P407 hydrogel formulation) (Wang et al., 2009; Piu et al., 2011). Similarly, a poloxamer formulation of the steroid triamcinolone acetonide yielded inner ear drug levels for at least 10 days (Honeder et al., 2014). Lee et al. (2004) reported that intratympanic administration of a poloxamer-based formulation of the antibiotic vancomycin successfully addressed MRSA infection in chronic otitis media. The pharmacokinetic profile can be altered by changing the nature and composition of the formulation, especially when non-aqueous soluble forms of a drug substance are used: insoluble forms of both dexamethasone and methylprednisolone, when formulated in a P407 hydrogel, yield significantly longer exposure in the inner ear than aqueous soluble forms (Wang et al., 2011). In all, an intricate interrelation exists between the pharmaceutical agent and the poloxamer copolymer. By modifying various parameters specific to the poloxamer hydrogel and bioactive molecule, a tailored drug release profile from a few days to several months can be developed, with a more homogenous basal-apical concentration gradient (Plontke et al., 2007b), relative to aqueous solutions. The addition of poloxamer to a PLGA-PEG-PLGA copolymer increased the inner ear exposure to the antiviral agent cidofovir while maintaining the absence of ototoxicity (Feng et al., 2014). Finally, poloxamers are listed on the FDA’s Generally Regarded As Safe (GRAS) list, and their administration is deemed safe in humans (Singh-Joy and McLain, 2008). While poloxamers are not biodegradable, their administration in the middle ear results in their rapid disappearance from that compartment within a couple of weeks, the elimination being a function of the poloxamer concentration (Engmer Berglin et al., 2015). In conditions where the middle ear was filled, intratympanic administration of poloxamer, but also other polymers, was associated with transient conductive hearing loss, consistent with the high viscosity of the polymers (Engmer Berglin et al., 2015). However, no evidence of toxicity was noted in the middle ear, cochlear and vestibular structures in preclinical studies when the dosing volume covered only the round window niche (Wang et al., 2011).

Poloxamer-based delivery systems have been evaluated in several neurotology clinical trials. OTIVIDEX (OTO-104, a micronized dexamethasone loaded P407 hydrogel formulation) is currently being developed for the treatment of Meniere’s disease. Findings from Phase 2 and 3 clinical studies reported that OTIVIDEX was well tolerated with no safety concerns identified (Lambert et al., 2012, 2016). A Phase 1 study conducted in healthy volunteers reported that OTO-311, a P407 based formulation of the non-competitive *N*-methyl-D-aspartate (NMDA) receptor antagonist gacyclidine being developed for the treatment of tinnitus, was well tolerated with no safety concerns identified (Anderson et al., 2019). Another poloxamer-based hydrogel formulation FX-322, a combinational therapy dubbed PCA (Progenitor Cell Activation), was evaluated in a Phase 1 clinical trial in adult patients with stable sensorineural loss scheduled for cochlear implantation. The investigators reported that FX-322 was well tolerated with no drug related adverse events. More recently a Phase 1/2 study of FX-322 in the same patient population completed enrollment, but to date no results have been reported. Finally, only a single poloxamer-based delivery system has been approved for otic delivery: OTIPRIO^^®^^ (an otic suspension of the fluoroquinolone antibacterial ciprofloxacin in poloxamer 407) is approved for two indications: (1) treatment of pediatric patients with bilateral otitis media with effusion undergoing tympanostomy tube placement and (2) acute otitis externa (Mair et al., 2015, 2016; Park et al., 2016; Dohar et al., 2018).

## Conclusion

Means to efficiently and reliably deliver therapeutic drugs to the inner ear compartment are being developed, with local delivery approaches now largely favored. In particular, a procedure that has been gaining acceptance in the physician’s practice is intratympanic injection. Combining intratympanic administration with the use of otic-specific formulations provides an effective approach to deliver reliable and sustained therapeutic levels to the inner ear. This approach has been tested in a number of neurotology clinical trials and several drugs employing this method are under development for the treatment of neurotology disorders.

## Author Contributions

Both authors contributed to the conception and designed the manuscript, revised the manuscript, read and approved the submitted version of the manuscript. FP wrote the first draft of the manuscript.

## Conflict of Interest Statement

The authors declare that the research was conducted in the absence of any commercial or financial relationships that could be construed as a potential conflict of interest.

## Footnotes

1 https://ir.aurismedical.com/news-releases/news-release-details/auris-medical-provides-business-update, press release March 13, 2018.
